# Typing of inflammatory lesions of the pituitary

**DOI:** 10.1007/s11102-021-01180-1

**Published:** 2021-08-31

**Authors:** J. Warmbier, D. K. Lüdecke, J. Flitsch, M. Buchfelder, R. Fahlbusch, U. J. Knappe, J. Kreutzer, R. Buslei, M. Bergmann, F. Heppner, M. Glatzel, W. Saeger

**Affiliations:** 1 Institute of Neuropathology of the University of Hamburg, UKE, 20246 Hamburg, Germany; 2 Clinic of Neurosurgery of the University of Hamburg, UKE, 20246 Hamburg, Germany; 3 Clinic of Neurosurgery, Friedrich-Alexander University Erlangen-Nürnberg (FAU), 91054 Erlangen, Germany; 4 International Neuroscience Institute (INI), Rudolf-Pichelmayr-Str. 4, 30625 Hannover, Germany; 5 Department of Neurosurgery, Johannes-Wesling-Klinikum Minden, Ruhr-University Bochum (RUB), 32429 Minden, Germany; 6 Praxis for Neurosurgery, 90941 Nuremberg, Germany; 7 Institute of Pathology, SozialStiftung Bamberg, 96049 Bamberg, Germany; 8 Institute of Neuropathology, Klinikum Bremen-Mitte, 28205 Bremen, Germany; 9 Institute of Neuropathology of the Humboldt University of Berlin, Charitè, 10117 Berlin, Germany; 10 Institutes of Pathology and Neuropathology of the University of Hamburg, UKE, Martinistraße 52, 20246 Hamburg, Germany

**Keywords:** Pituitary, Hypophysitis, Pathology, Classification

## Abstract

Inflammatory pituitary lesions account for 1.8% of all specimens from the German Pituitary Tumor Registry. They occure in 0.5% of the autoptical specimens and in 2.2% of the surgical cases. Women are significantly more often affected than men and are often younger when first diagnosed. In general, primary and secondary inflammation can be distinguished, with secondary types occurring more frequently (75.1%) than idiopathic inflammatory lesions (15.4%). In primary inflammation, the lymphocytic type is more common (88.5%) than the granulomatous type of hypophysitis (11.5%). The most common causes of secondary inflammation are Rathke’s cleft cysts (48.6%), followed by tumors (17.4%) such as the craniopharyngioma (9.1%), adenoma (5.5%) or germinoma (2.0%). More causes are tumor-like lesions (7.1%) such as xanthogranuloma (3.5%) or Langerhans histiocytosis (3.5%), abscesses (5.5%), generalized infections (5.1%), spreaded inflammations (4.7%) and previous surgeries (4.0%). In 1.6% of all specimens the reason for the inflammation remains unclear. The described classification of hypophysitis is important for specific treatment planning after surgery.

## Introduction

**Fig. 1:**
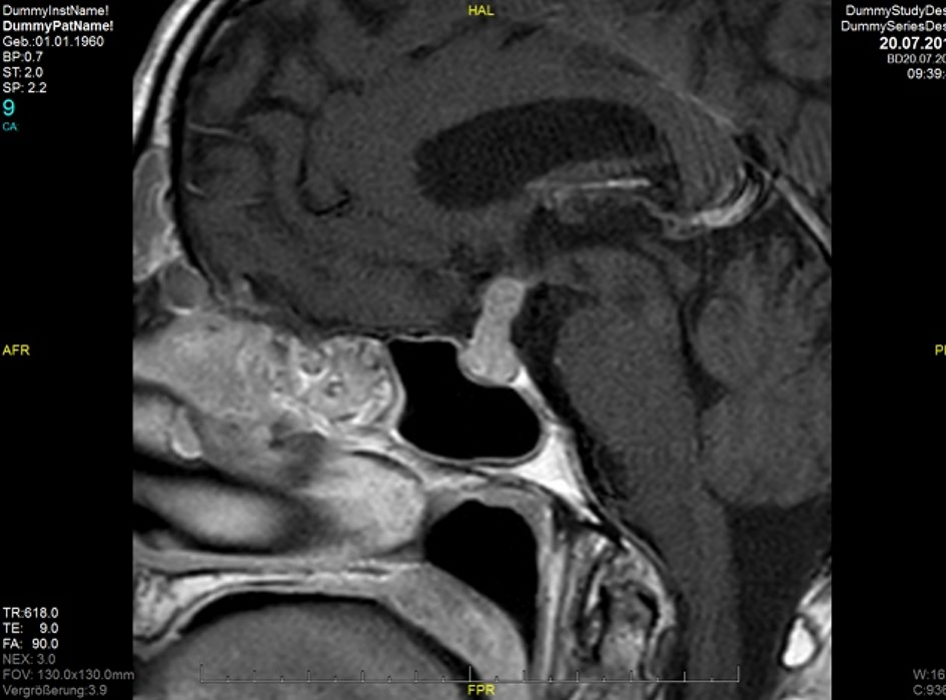
MRI image of typical lymphocytic hypopyhsitis with stalk involvement

Inflammatory findings in the pituitary gland represent a rare differential diagnosis to neoplastic lesions of the sellar region. Since clinical symptoms, radiological imaging (Fig. 1) and sometimes even histological findings are often non-specific, a precise diagnosis can be difficult and only be done by comparisons of radiology, pathology and clinical data [1]. Small accumulations of lymphocytes—occasionally detected in the pituitary intermediate zone—should not be taken into account as they are not an expression of inflammation, but rather represent residues of lymphoid tissue from the craniopharyngeal duct [2]. Primary idiopathic hypophysitis is to be differentiated from secondary types such as inflammatory lesions due to Rathke cleft cysts, craniopharyngeomas or germinomas or involvement of the pituitary in generalized inflammation (e.g. sarcoidosis, IgG4-related disease) [3].

An autoimmune genesis is assumed in primary hypophysitis, but so far no antipituitary antibodies have been determined whose specificity and sensitivity are high enough to allow an accurate diagnosis [4]. Future research remains necessary for further categorizing autoimmune hypophysitis [5]. Recently an inflammatory type was described that develops from autoimmunity against the transcription factor PIT-1 [6].

Some classifications published in the literature [3–5, 7–10] present other principles, differentiating primary hypophysitis by the cellular type of inflammation. Some only describe lymphocytic and granulomatous hypophysitis as primary lesions [3], others define also xanthomatous hypophysitis as primary [7, 9], and some also include IgG4-associated hypophysitis and necrotizing hypophysitis as primary inflammations [4, 5, 10]. Furthermore, mixed types such as xanthogranulomatous hypophysitis and drug-induced hypophysitis (due to immune checkpoint inhibitors) can be found as independent entities in classifications [8].

The aim of our study was to propose a pathogenetic classification based on our very large collection of specimens in the German Registry of Pituitary Tumors that contains not only tumorous but also inflammatory pseudo-tumorous lesions. On the one hand, the focus was on emphasizing the importance of differentiating the various types of lesions, on the other hand, to point out the importance of IgG4-associated inflammation in this context. Since the study is retrospective, many nowadays necessary clinical data could not be included in contrast to prospective studies with smaller number of cases. However, we would like to emphasize the large number of patients in our study, as we are not aware of any other study on this topic that has included so many cases. The collection of Caturegli et al. [7] comprehends 379 patients but based on metastudies partly without histopathology. Therefore, this publication is not completely comparable with our collection.

## Material and methods

For our studies, the collective was extracted from the files of the personal collection of one of the authors (WS) of the years 1970 to 1995 and the data from the German Pituitary Tumor Registry of the German Society for Endocrinology since its beginning in 1995 up to 2018. In the following the cases from 1970 to 2018 are summarized under the term of German Pituitary Tumor Registry.

Specimens had been embedded in paraffin. Sections were stained with hematoxylin–eosin and PAS and immunostained for pituitary hormones. Special cases were also immunostained for differentiating inflammatory infiltrates (CD3, CD5, CD20, CD45, CD68. IgG4, CD138, CD1a).

All cases with inflammatory infiltrates described in the histological reports were extracted from the database and sorted according to their diagnosis. If the diagnosis was inconclusive, clinical files were requested and examined for further indications regarding the diagnosis, such as subsequent tests, specific clinical symptoms or recurrences with new findings.

It should also be emphasized that according to Casanueva et al. a close collaboration between pathologists, endocrinologists, neurosurgeons and radiologists defines the criteria for a pituitary tumor center of excellence (PTCOE) [11]. Such cooperation formed the basis for the diagnoses and thus for the typing presented.

## Histological criteria for different inflammatory types

**Fig. 2:**
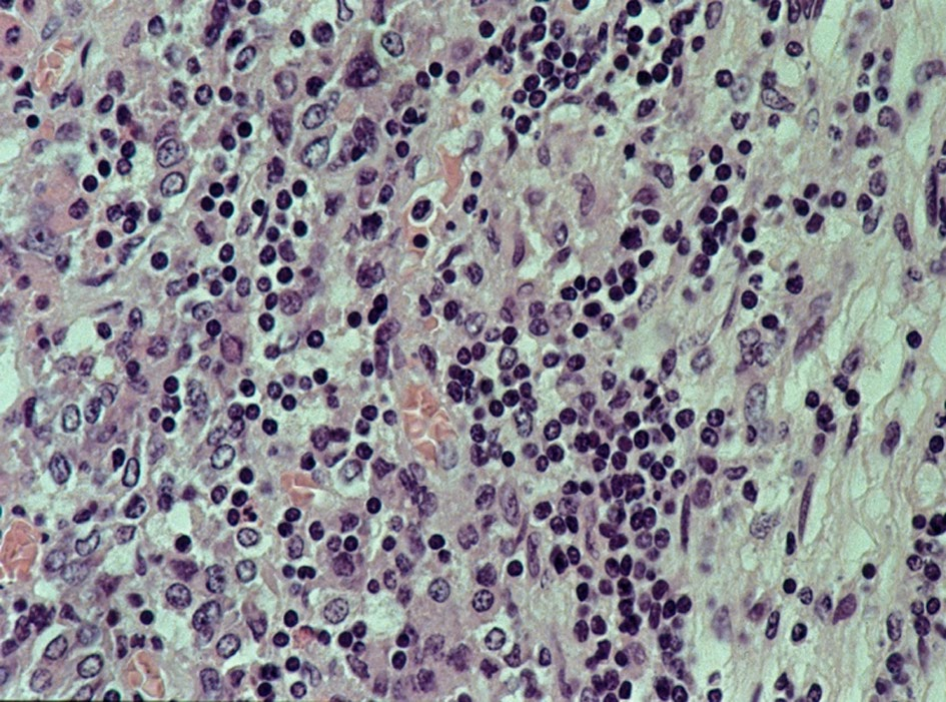
Lymphocytic hypophysitis: partly focal, partly diffuse lymphocytic infiltration of the anterior lobe. HE staining, magnification ×400

**Fig. 3:**
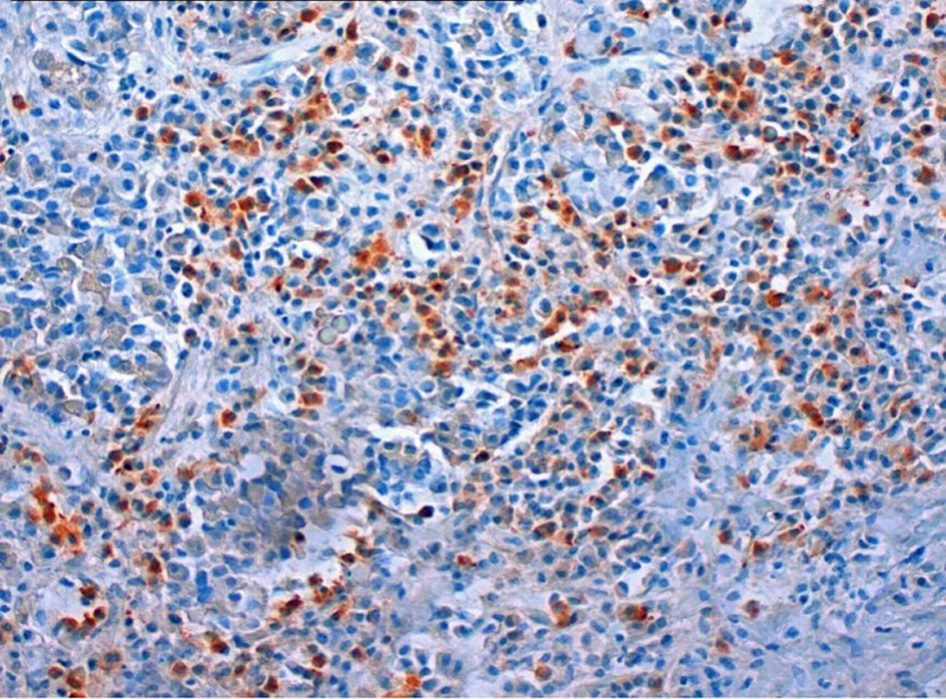
Lymphocytic hypophysitis of IgG4-type: many IgG4-positive lymphocytes. IgG4-immunostaining, magnification ×250

We used the following criteria as indicators of inflammation: lympho-plasmacellular infiltrates of varying density (Fig. 2), granulocytes, granulation tissue, fibroses, scars with slight inflammatory infiltrates, IgG4-positivity in plasma cells (Fig. 3) and serum IgG4 levels.

The type and extent of the cellular infiltrate determined our diagnosis. A diffuse distribution pattern of the infiltrating lymphocytes and plasmocytes suggests an autoimmune primary hypophysitis, a more focal pattern for a secondary origin. If predominantly lymphocytic infiltrates are present, primary inflammation can be assumed; mixed-cell infiltrates consisting of lymphocytes, plasma cells, granulocytes, and histiocytes indicate secondary hypophysitis. A dense lymphoplasmacytic infiltrate with a percentage of IgG4‐positive of all IgG‐positive cells exceeding 40% and absolute numbers of IgG4‐positive plasma cells exceeding at least 10 per high power field (HPF) (Fig. 3), accompanied by a fibrosis and obliterative phlebitis suggest the diagnosis of an IgG4-related disease [12].

**Fig. 4:**
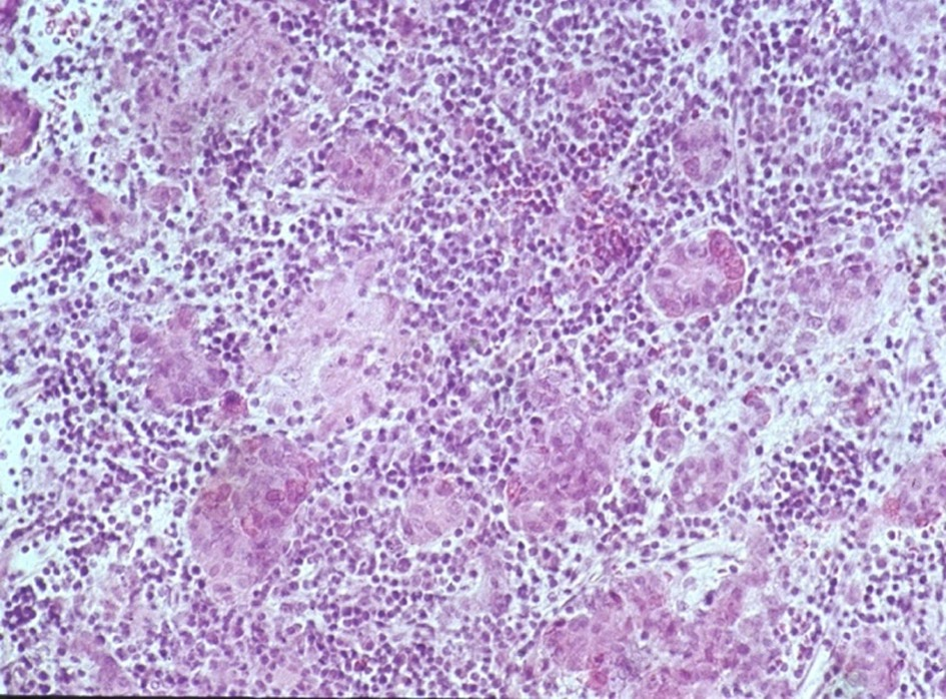
Granulomatous hypophysitis: lymphocytic infiltration of the anterior lobe and granulomas with epitheloid cells and giant cells. HE staining, magnification ×250

**Fig. 5:**
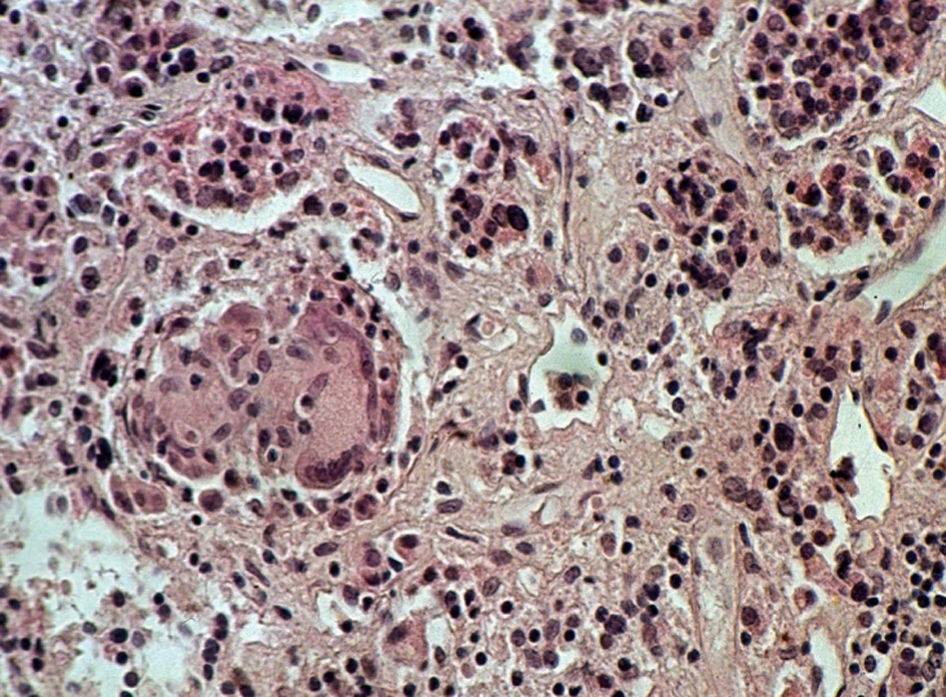
Granulomatous hypophysitis: granulomas with multinucleated giant cells and sparse lymphocytic infiltration. HE staining, magnification ×440

If a secondary inflammation could not be ruled out with certainty, or if the lymphocytic distribution was unusual, these cases were classified as probably primary lymphocytic hypophysitis. The diagnosis of definitely primary granulomatous hypophysitis (Figs. 4 and 5) can only be made if a generalized granulomatous infection, such as sarcoidosis or tuberculosis, has been ruled out [3]. This always requires reliable clinical information. If these were not available, then those patient cases were assigned to the category of probably primary granulomatous hypophysitis. It should be noted that cases were also reported in which lymphocytic hypophysitis cannot be reliably differentiated from granulomatous hypophysitis [13].

The inflammations caused by another lesion were grouped under the category of secondary hypophysitis and subdivided according to their causal lesion. The difference between a hematogenously spreaded inflammation and a hypophysitis due to generalized inflammation hereby is that the hematogenous form originated from a former sepsis without any other active foci at this point.

If there was a case that could no longer be reliably assessed and also showed another lesion nearby, or if there was an atypical lesion that was structurally similar to a secondary inflammation, these were classified as likely secondary inflammation. If there was an uncharacteristic inflammatory lesion for which no evidence of a primary or secondary etiology was found, or if the specimens were insufficient or surrounding non-inflammatory tissue was lacking a clear diagnosis was not possible, these cases were classified as unclear hypophysitis.

In our collection, there was no case of drug-induced hypophysitis. This is why this category is not reflected in our classification. Nevertheless, it should be emphasized that this form of disease caused by checkpoint inhibitors is nowadays to be regarded as an important differential diagnosis [14].

## Results

From 1970 to 2018 18,329 cases were collected in the Pituitary Tumor Registry. Inflammatory pituitary masses were histologically detected in 1.8% (n = 337) of all specimens of the sellar region. They occurred for 0.5% of the autoptical specimens and for 2.2% of the surgical cases. Women were significantly more often affected by hypophysitis than men (n = 225 vs. n = 112) and were often younger when first diagnosed (Table 1). Primary and secondary inflammation can be distinguished, with secondary types occurring more frequently (77.2%) than idiopathic inflammatory lesions (20.7%) (Table 2).

**Table 1:** Hypophysitis gender comparison

	Amount	Average age at presentation (years, mean ± SD)	Minimum age at presentation (years)	Maximum age at presentation (years)
Women	225	38.9 ± 18.1	0	89
Men	112	42.2 ± 19.9	4	98
Total	337	40.0 ± 18.8	0	98

**Table 2:** Hypophysitis classification I

	Certainly primary hypophyitis	Probably primary hypophysitis	Certainly secondary hypophysitis	Probably secondary hypophysitis	Not clear
Amount	52	18	253	7	7
% of hypophysitis	15.4	5.3	75.1	2.1	2.1
% in total	0.28	0.1	1.38	0.04	0.04

### Certainly primary hypophysitis

#### Lymphocytic and granulomatous hypophysitis

52 patient cases are classified as certainly primary hypophysitis (15.4% of all inflammatory diagnoses) (Fig. 2). Within these the lymphocytic form is significantly more common (88.5%) than the granulomatous hypophysitis (11.5%) (Figs. 4 and 5). 59.6% of all patients with certainly primary hypophysitis are woman, 40.4% are men (Table 3). The average age of the patients with definitely primary lymphocytic hypophysitis is 43.8 years, the average age of women is 39.0 years and the one of men 50.7 years.

**Table 3:** Primary hypophysitis

	Lymphocytic hypophsitis	Granulomatous hypophysitis	Total
Amount	n = 46	n = 6	n = 52
%	88.5%	11.5%	75.1%
Women	n = 27	n = 4	n = 31 (59.6%)
Men	n = 19	n = 2	n = 21 (40.4%)

#### IgG4-related hypophysitis

13 of 21 re-examined cases in our database (61.9%) previously diagnosed as primary hypophysitis fulfill the criteria for IgG4-related disease (Fig. 3). The positive results occur in six male patients (46.2%) and in seven female patients (53.8%). The overall mean age is 48.0 years, that of women 43.4 years and that of men 53.3 years.

### Probably primary hypophysitis

In the cases without clear diagnosis, but with probably primary nature of inflammation, the percentage of granulomatous inflammations (2.7%) is the same as that of lymphocytic inflammations (2.7%). The total number of these patient cases was 18.

### Certainly secondary hypophysitis

The diagnosis of definitely secondary hypophysitis could be determined in 253 cases (75.1%). The causes of this inflammation are the following (Table 4).

**Table 4:** Secondary Hypophysitis

	Amount	% in certainly secondary hypophysitis	% in total
Cysts	138	54.5	40.9
Spreaded inflammations	12	4.7	3.6
Tumor-associtaed lesions	44	17.1	13.1
Tumor-like lesions	18	7.1	5.3
Generalized inflammations	13	5.1	3.9
Post-surgery	10	4.0	3.0
Abcesses	14	5.5	4.2
Not classified	4	1.6	1.2
Total	253	100	75.1

#### Cyst-induced inflammation

**Fig. 6:**
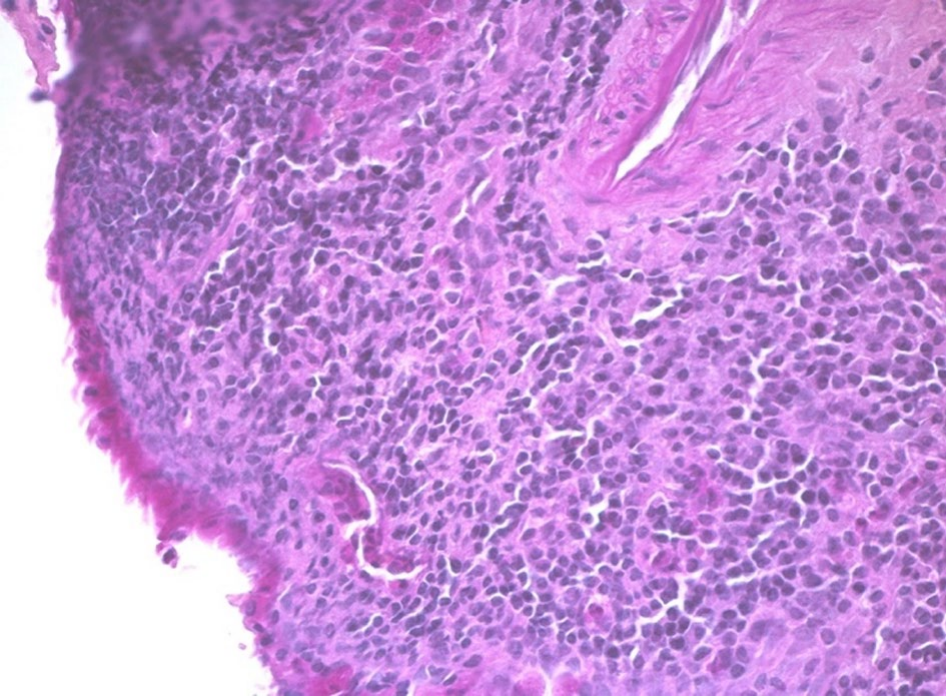
Rathke’s cleft cyst: lining of cubic and cylindrical epithelium partly with cilia, surrounded by lymphocytic infiltration. PAS staining, magnification ×250

Cyst-induced inflammation is diagnosed in 138 cases (40.9%). Rathke`s cleft cysts are the most common type of cysts and the most common type for secondary inflammation (37.2%) (Fig. 6). They occurred significantly more often in women.

#### Spreaded inflammation and abcesses

**Fig. 7:**
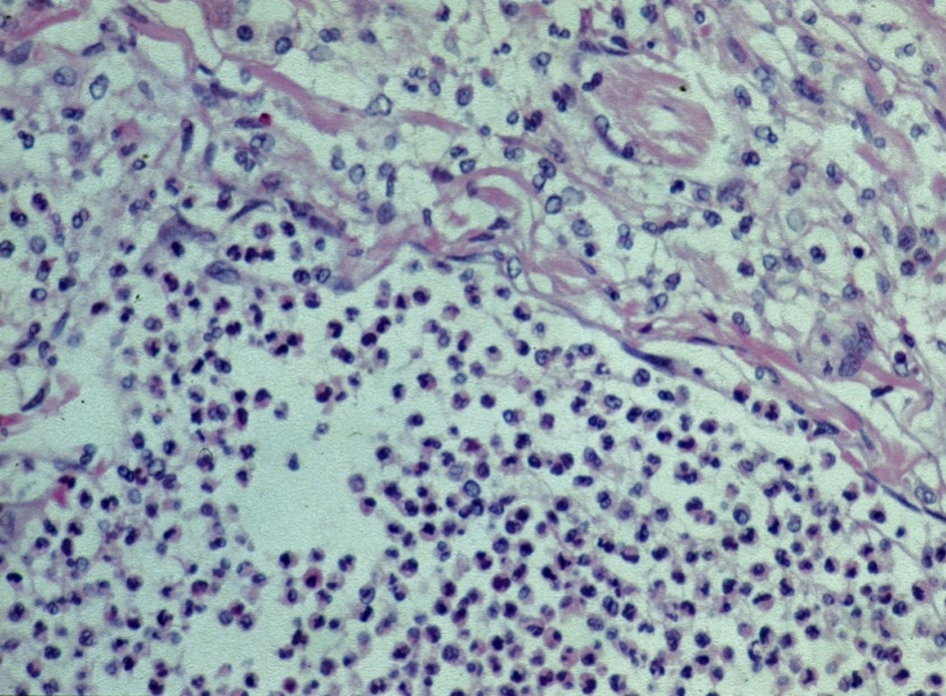
Acute granulocytic inflammation as first stage of abscess surrounded by adenohypophysis with sparse inflammation, no fibrous tissue. PAS staining, magnification ×440

**Fig. 8:**
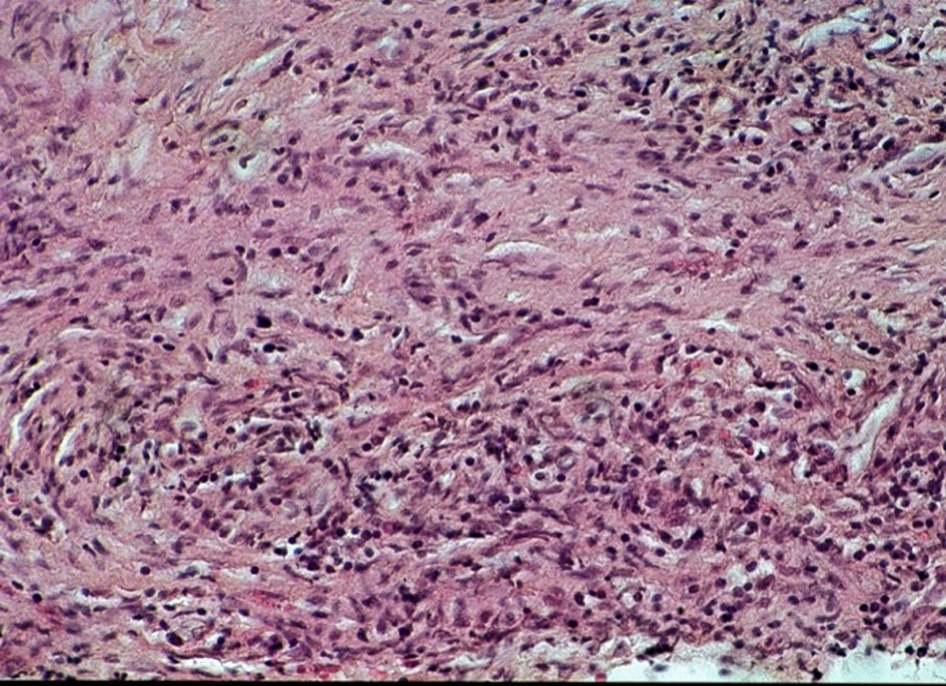
Chronic inflammation with old fibrous abscess wall. HE staining, magnification ×250

Twelve inflammatory lesions (3.6%) were due to a spreaded inflammation,

4.2% (n = 14) of the definitely secondary hypophysitis developed due to an abscess (Figs. 7 and 8), the cause of which could no longer be clearly determined.

#### Tumor-induced hypophysitis

**Fig. 9:**
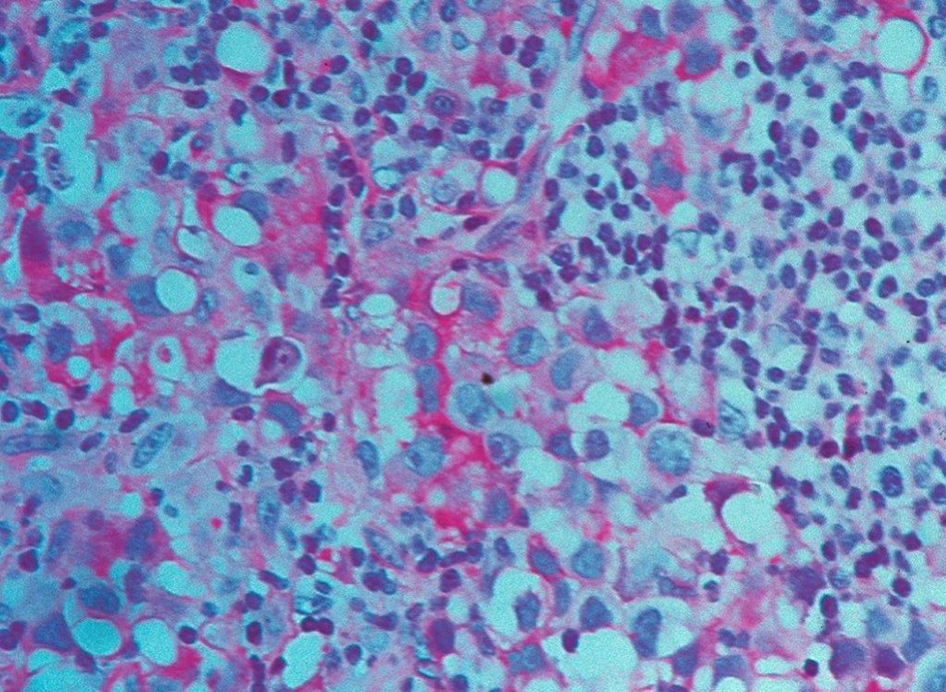
Germinoma; lymphocytic infiltrates within the tumor tissue. PAS staining, magnification ×480

13.1% (n = 44) of the patients developed hypophysitis due to a tumor. Craniopharyngiomas are the most likely tumorous lesions leading to inflammatory reactions of the pituitary (52.3%), followed by adenoma (31.8%) and germinoma (11.4%) (Table 5) (Fig. 9). 60% of the patients with germinoma were 12 years old or younger and the female to male ratio was 4:1.

**Table 5:** Tumorous lesions

	Germinoma	Craniopharyngioma	Adenoma	Metastasis of squamous cell carcinoma	Spindle cell oncocytoma	Total
Amount	n = 5	n = 23	n = 14	n = 1	n = 1	n = 44
% in tumor-associated inflammations	11.4%	52.3%	31.8%	2.3%	2.3%	100%
% in total	1.5%	6.8%	4.2%	0.3%	0.3%	13.2%

In the group of adenomas the following subtypes were recorded: two gonadotrophic adenomas, one acidophilic stem cell adenoma (0.3%), two ACTH cell adenomas (0.6%), two null cell adenomas (0.6%), five prolactin cell adenomas (1.5%), one mixed GH / prolactin cell adenoma (0.3%) and one GH cell adenoma (0.3%).

#### Tumor-like lesions

Xanthogranuloma or xanthomatous inflammation and Langerhans cell histiocytosis led to concomitant inflammation in 18 patients (5.3%).

#### Generalized inflammation

3.9% of all cases with inflammatory pituitary lesions have been due to generalized inflammations such as HIV/AIDS (0.9%), tuberculosis (1.2%), herpes simplex (0.6%), sarcoidosis (0.9%) or Crohn’s disease (0.3%) (Table 6).

**Table 6:** Generalized inflammations

	M. Crohn	Herpes simplex	AIDS	Sarcoidosis	Tuberculosis	In total
Amount	n = 1	n = 2	n = 3	n = 3	n = 4	13
% in total	0.3	0.6	0.9	0.9	1.2	3.9

#### Pre-operation

Inflammatory reactions due to a previous surgical procedure were present in 3.0% of the patient cases (n = 10).

#### Not classified

In 1.2% of all cases (n = 4) from this category, the cause of the secondary inflammation could not be reliably classified.

### Probably secondary hypophysitis

Secondary hypophysitis was considered probable in seven cases (2.1%), but a primary etiology could not be excluded with certainty. These cases included two that could not be further classified (0.6%) and one each with a cyst, an abscess of unknown cause, locally extended inflammation, tumor-associated inflammation, and a tumor-like lesion (0.3%).

### Uncertain cases

A clear classification of the inflammatory lesion was not possible in all these patients. There was a total of seven cases (2.1%) in which the etiology of the inflammation could not be determined. In one case, the classification failed due to insufficient specimens (0.3%). In the remaining six cases, no diagnosis could be made due to unclassifiable features (1.8%).

## Discussion

The differentiation of inflammatory and tumor-like lesions in the sellar region is very important for the patients. A clear understanding of the inflammation origin through the histopathological diagnosis offers different therapeutic approaches (Table  7).

**Table 7:**
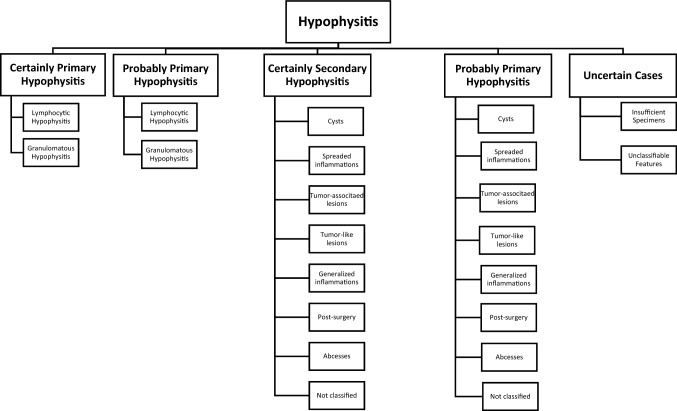
Hypophysitis classification II

Clinicopathologic studies on hypophysitis are published mostly in case reports or very small series. The paper of Tashiro et al. [15] comprehends 31 patients with very different endocrine diseases or pregnancies. To the best of our knowledge, our histopathologic collection of 337 cases of hypophysitis is the largest of all published series so far.

In a collection of surgical specimens patients suffered from primary hypophysitis in 0.38 to 1.1% of cases [16] whereas in our collection between 1970 and 2018, the diagnosis of certainly primary hypophysitis is made in 0.32% of surgical specimens and a probable primary hypophysitis in 0.11%. Both groups together add up to 0.43%, therefore the same range as published [16].

The IgG4-associated hypophysitis represents an important differential diagnosis of growing clinical interest. Inflammatory lesions previously suspected to occur as a primary local lesion could thus have been part of a generalized disease.

In our collection we found that in 13 of 29 cases an inflammation previously diagnosed as primary hypophysitis met the criteria for an IgG4-associated disease and the first diagnosis of idiopathic primary hypophysitis must be revised [12]. There were cases with the involvement of at least one other organ. The average age of patients was 62 years. If no other organs were known to be involved at the time of the histological diagnosis, the average age was 49 years [17–26].

Pituitary involvement in generalized IgG4-associated inflammation was very rare (0–8%) in other studies [12, 27–31]. The number of IgG4-associated hypophysitis without further organ involvement was 65%. This leads to the assumption of a separate entity of hypophysitis independently of the generalized IgG4-associated inflammation [12].

From 2000 to 2018, 33 cases in our collective were tested for IgG4-positive plasma cells, of which 13 cases (39.4%) tested positive. If we examine the entire group of primary hypophysitis (N = 55) in the period from 2000 onwards, the percentage of IgG4-positivity is 23.6%, with 61.8% of the cases with a primary form of inflammation not been tested for IgG4 -positive plasma cells.

Excluding the untested cases, the percentage of IgG4-positive test results in previously diagnosed primary hypophysitis is 61.9%. This frequency being significantly higher than previously published [12, 17, 25], is probably related to the large number of untested cases, but it shows that IgG4-associated hypophysitis is probably a frequent type of pituitary inflammation.

In our collective there is a slight predominance of female patients with IgG4-positive test results (53.8%). The overall average age of 48 years is slightly below that reported by Li et al. published [32], whereby we also observe that men are on average older at diagnosis (53.3 years vs. 43.4 years).

Some authors suspect that granulomatous and lymphocytic hypophysitis are the same entity [15, 33–37]. One reason is the existence of structural similarities between the two forms of inflammation. Degranulated endocrine cells, focal oncocytic changes in the secretory cells and inflammatory cells within the periacinar membrane were detected in both lesions [15, 34, 37]. Since the occurrence of granulomatous hypophysitis is in some cases also accompanied by other autoimmune diseases, such as Hashimoto's thyroiditis, an autoimmune pathogenesis is also assumed. Honegger et al. describe an incidence of 22% for coexisting autoimmune diseases [35]. This thesis is also supported by the fact that in both lymphocytic and granulomatous hypophysitis contain immunoreactive antigens against macrophages in the cytoplasm of the giant cells [15, 34–37].

Secondary hypophysitis is an accompanying inflammation as a reaction to an evaluable lesion [1]. In our collective Rathke cleft cysts are the most common lesion causing secondary inflammation. Similar results were published by Teramoto et al. [38]. In 37.2% of all secondary inflammations based on Rathke’s cleft cyst, in further 2.4% Rathke cleft cyst is assumed. The reason for the inflammatory reaction of the surrounding tissue in our opinion lies in leakage of cyst fluid due to disrupture of the cyst wall. In most studies on collectives of Rathke cysts, an excess of females was observed [39–41]. In our collective, the female proportion of patients with Rathke’s cleft cysts is significantly higher than the male and is 74.8% in the group with a certain diagnosis. It could be proven that there was a connection between the severity of secondary hypophysitis and the occurrence of clinical symptoms, which reinforces the importance of diagnosing an inflammatory component in connection with Rathke cleft cysts [42].

Infections in other organs can be spreaded to the pituitary gland hematogenously or via nearby lesions [43, 44]. Abscesses can develop from pituitary lesions such as cysts and tumors. In 50% to 70% of all cases, no cause for the abscess development can be found [45]. Furthermore in about 50% of the cases the causative pathogenic microorganisms cannot be isolated [46]. In 1965 Montrieul et al. put forward the thesis that abscesses can also arise sterile through aseptic necrosis of pituitary tumors, since in some cases no bacterial populations could be detected in the abscess content [47]. Other authors believe that the “sterile” exudate is due to either inadequate bacterial tests for anaerobic microorganisms or previous antibiotic therapy [46, 48]. The incidence of pituitary abscesses is generally given as less than 1% (0.2–1%) of pituitary lesions [45]. The German Pituitary Tumor Registry comprises 18,329 cases. Of these, a pituitary abscess was diagnosed in 14 cases (0.01%). Despite the low incidence rate, pituitary abscesses should be considered as a differential diagnosis, as the earlier figures make it clear that incorrect or missing therapy leads to a high mortality rate [46, 48–50].

Since Sautner et al. introduced the term of secondary hypophysitis [51], many cases of tumor-induced secondary hypophysitis have been described. Adenomas, craniopharyngiomas and germinomas, among others, can give rise to inflammation [10].

In our database, an adenoma is the cause of inflammation in 4.2% of all cases with hypophysitis. All types of adenoma occur, with prolactin adenomas being the most common (35.7%). In 1983, Holck et al. discussed different causes for inflammations due to adenoma [52]. The original theories included the coincidental occurrence of two independent lesions, the triggering of inflammation by the adenoma through secretion of certain substances, or a reaction of the non-neoplastic tissue to the adenoma through compression or foreign body reaction. These theories are also described in more recent publications, although the adenoma antigens for inflammatory reaction could not yet be fully determined [53, 54]. In 2020, Yamamoto et al. found out that one antigen being expressed by neoplasms is associated with pituitary diseases—the pituitary-specific transcription factor PIT-1 [6].

Adamantinomatous craniopharyngiomas are characterized by stratified squamous epithelium with a palisade-like basal cell layer and sometimes inflammatory infiltrations. Rathke cysts can also be lined by squamous epithelium, some of which also have keratinization. Necrosis of the squamous epithelium might trigger the inflammation. Infiltrates in the cyst walls have been described, especially in connection with relapses. In addition, typical craniopharyngioma findings such as calcifications, hemosiderin deposits and cholesterol crystals were observed in Rathke cysts which sometimes makes it difficult to distinguish them from each other.

Another pituitary neoplasm that can lead to inflammatory infiltrates is germinoma, which according to the WHO is the most common intra- and suprasellar germ cell tumor and occurs mainly in humans in the first two decades of life [55]. In our analysis, germinomas are present in 11.4% of tumor-associated inflammations. These occur in 60% of cases in prepubertal children. The diagnosis of a germinoma can be difficult because lymphocytic infiltrates are usually present in different amounts within this tumor [55]. Since these lymphocytes often infiltrate not only the tumor but also surrounding tissues and the number of lymphocytes may be very high, tumor cells cannot always clearly be identified in biopsies and the diagnosis of hypophysitis may be a pitfall [56, 57]. It should be emphasized here that it is precisely the clinical symptoms and radiological changes that are present due to the inflammation that provide the first important clues in the diagnosis of a germinoma. Therefore, a germinoma diagnosis can only be made by combining histopathological, clinical and radiological findings.

Some authors define the xanthomatous hypophysitis as primary lesion [7, 9]. This assumption contradicts the observation that the inflammatory infiltrate occurs locally and the surrounding adenohypophysis appears unaffected [58, 59]. Nowadays, xanthogranulomatous hypophysitis is thought to be the result of inflammations and foreign body giant cells as reaction to necrotic squamous epithelia [60], and therefore we classified it as a secondary developed tumor-like lesion.

Generalized inflammatory diseases of other organs such as sarcoidosis, Wegener's disease, Crohn's disease and Sjögren's syndrome or generalized viral/bacterial infections can also affect the pituitary gland and trigger an inflammatory reaction [1]. Sarcoidosis is often undiagnosed during a lifetime. An autopsy on a large group revealed a sarcoid diagnosis in 43 people, with the disease ante-mortem only known in three cases [61]. A comparable observation was also described in a recent study with regard to neurosarcoidosis. The percentage of clinically diagnosed neurosarcoidosis (5–13%) was below that of post-mortem cases (14–50%) [62]. Because histopathology of sarcoidosis and idiopathic granulomatous hypophysitis is identical, the latter can only be diagnosed after a sarcoid disease has been ruled out [3].

The human immunodeficiency virus (HIV) is the viral infection that most commonly leads to a pituitary inflammation in up to 23% of patients suffering from AIDS [63].

Any other viral inflammation of the brain can also lead to an inflammatory reaction of the pituitary gland [10]. In about 0.01 to 0.02% of patients with a herpes simplex infection, the virus enters the central nervous system via the olfactory bulb and causes a herpes simplex encephalitis. This leads to death in over 70% of untreated patients [64].

Pituitary inflammations due to bacterial infection are associated, for example, with the mycobacterium tuberculosis complex or the bacterium Treponema pallidum [10]. It is believed that the pituitary gland is involved in up to 49% of patients with tuberculosis [65]. In only one case of our collective the diagnosis was confirmed by autopsy. In the other three cases, tuberculosis was suspected. One patient was found to have tuberculous spondylitis at a later date. Nine cases of granulomatous inflammation were found in the group of probably primary hypophysitis. It cannot be ruled out that these cases could also be a reaction to a tuberculosis infection.

Correct classification of pituitary lesions is the key to proper treatment. For clinicians the diagnosis of hypophysitis after pituitary surgery may be unexpected. However, generally established therapy regimens for hypophysitis do not exist yet [34]. Therefore further interdisciplinary treatment should involve an endocrinologist, as was proposed for pituitary tumor centers [11], and a center-specific regimen should be established in any neurooncologic center.

Inflammation of the pituitary is an important differential diagnosis, requiring sophisticated histological examination. Since the incidence of such lesions is low, it seems to be advisable to involve a specialized pathology unit into the diagnostic work-up of such (unexpected) lesions. In Germany, this is facilitated by the pituitary tumor registry of the German Endocrinological Society (DGE). It should be noted that the histopathology of inflammation provides important information about its pathogenesis. Our study aims to provide a well substantiated insight into the different morphology of the inflammation. The proposed typing of inflammatory lesions on the one hand may help clinicians to understand the etiology of the inflammation in a given case and therefore direct to further diagnostic steps and patient-specific therapy. On the other hand it may enable further studies to establish generally accepted therapy regimen.
